# Adsorption Kinetics of Chromium (VI) from Aqueous Solution Using Agroindustrial Waste-Based Biochars Derived from Orange Peels and Peanut Shells

**DOI:** 10.3390/polym18141793

**Published:** 2026-07-22

**Authors:** Adrian Ferrucio Garcia-Morales, Oscar Eduardo Ortiz-Contreras, Alejandra Álvarez-López, Vanessa Vallejo-Becerra, Juan Campos-Guillén, Miguel Angel Ramos-López, Mónica López-Velarde Santos, Ricardo Chaparro-Sánchez, Sarai E. Favela-Camacho, Oscar Yael Barrón-García, José Alberto Rodríguez-Morales, Aldo Amaro-Reyes

**Affiliations:** 1 Faculty of Engineering, Autonomous University of Querétaro, Cerro de las Campanas S/N, Las Campanas, Queretaro 76010, Mexico; agarcia89@alumnos.uaq.mx (A.F.G.-M.); oortiz13@alumnos.uaq.mx (O.E.O.-C.);; 2 Research Center for Semiconductor Materials, Sustainability and Renewable Energy (CIMSSER), Faculty of Chemistry, Autonomous University of Queretaro, Cerro de las Campanas S/N, Las Campanas, Queretaro 76010, Mexico; 3 Faculty of Informatics, Autonomous University of Querétaro, Av. de las Ciencias S/N, Juriquilla 76230, Mexico; 4 Institute of Engineering and Technology, Autonomous University of Juarez City, Avenida del Charro S/N y Calle Henry Dunant, Omega, Juarez City 32584, Mexico; 5 Research and Graduate Studies in Food Science, School of Chemistry, Autonomous University of Queretaro, Cerro de las Campanas S/N, Las Campanas, Queretaro 76010, Mexico; 6 División Industrial, Universidad Tecnológica de Querétaro, Av. Pie de la Cuesta 2501, Nacional, Queretaro 76148, Mexico

**Keywords:** adsorption, agricultural waste, biochar, hexavalent chromium, kinetics

## Abstract

Hexavalent chromium (Cr(VI)) is a highly toxic, non-biodegradable, and carcinogenic heavy metal. Its continuous release into aquatic ecosystems demands efficient, low-cost adsorbents. In this study, orange peel and peanut shell residues were thermally modified at 250 °C to enhance Cr(VI) remediation. Structural characterization confirmed that low-temperature calcination transforms raw agroindustrial wastes into functional biochars with a chemical architecture primed for cooperative Cr(VI) removal. N_2_ physisorption revealed a hierarchical mesoporous network with average pore diameters of 30.6 nm (calcined orange peel) and 15.4 nm (calcined peanut shell), despite low specific surface areas. Batch adsorption experiments demonstrated that removal kinetics reached equilibrium within 5 min for the modified biochars. Isotherm modeling showed that the adsorption process was best described by the Freundlich and Sips models. The calculated Sips heterogeneity factors (*β_S_* > 1) provided evidence of a cooperative multi-layer adsorption mechanism, attributed to the induced mesoporosity: initial chemisorption at high-energy sites facilitates the continuous anchoring of additional Cr(VI) ions without premature saturation. Ultimately, this study demonstrates that low-temperature calcination is a viable strategy to transform agricultural waste into kinetically efficient, cooperative adsorbents for wastewater treatment.

## 1. Introduction

Economic growth and industrial development have increased wastewater contamination, primarily by heavy metals such as Pb, Zn, Ni, Cr, and Hg, which are highly toxic and non-biodegradable [1,2,3]. Hexavalent chromium [Cr(VI)] is considered one of the most dangerous heavy metal pollutants in aquatic ecosystems. Unlike its trivalent counterpart [Cr(III)], which is relatively insoluble and poorly permeable to biological membranes, Cr(VI) exists primarily as highly soluble oxyanions that easily mimic essential nutrients like sulfate. This structural mimicry allows Cr(VI) to readily cross cell membranes. Once intracellular, it undergoes a reduction to Cr(III), a process that generates massive oxidative stress and DNA damage, leading to severe mutations and several types of cancer [4,5]. The main sources of chromium pollution include mining, leather tanning, cement industry, dyes, and steel production [6].

Numerous physical, chemical, and biological methods are used to remove Cr(VI) from water; however, filtration-assisted adsorption has been one of the most efficient and cost-effective options in recent decades [7,8,9]. This process is attractive due to its low cost, simplicity of design and operation, as well as its effectiveness in the removal of pollutants [10]. While activated carbon, chitosan, zeolites, and graphene oxides are the most widely used adsorbents, their high costs have driven the search for more economical and eco-friendly options such as agricultural waste, i.e., bioadsorbents [7,11,12,13,14].

Approximately one-third of all food produced (1.3 billion tons annually) is wasted, causing significant impacts on soil and aquifers [13]. In this context, valorizing agroindustrial residues becomes a pressing environmental priority.

Orange peels and peanut shells are here defined as secondary agroindustrial by-products—residues resulting from the industrial transformation of primary crops [13]. These abundant biomass residues have high potential for Cr(VI) removal in wastewater due to their cellulose, hemicellulose, and lignin content, which possess functional groups capable of sequestering these metal species [15,16]. Orange represents 75% of citrus fruits globally, and yet in Mexico, out of an annual production of approximately 4.9 million tons, an estimated 17% is lost or wasted along the supply chain, representing an abundant source of underutilized biomass [17,18]. Globally, its production generates approximately 32 million tons of peel as waste [19]. Due to its composition of carboxyl and hydroxyl functional groups, orange peel is a promising raw material for Cr(VI) decontamination processes [20].

On the other hand, peanut shells constitute approximately 22% of the fruit’s weight, generating over 10 million tons of waste annually [21]. These shells possess a layered structure (pericarp, exocarp, mesocarp, and endocarp), rich in cellulosic compounds containing hydroxyl and carboxyl functional groups useful for Cr(VI) adsorption, making them an economical and viable bio-based material for environmental treatments [22].

To evaluate the efficiency of such materials, adsorption kinetics are employed to describe how the amount of adsorbate on the surface varies over time. This process is influenced by factors such as adsorbate concentration, temperature, adsorbent surface area, and specific adsorbent–adsorbate interactions [23,24]. The study of kinetics helps to determine the adsorption mechanism and allows the establishment of mathematical models such as pseudo-first- or pseudo-second-order, which describe how the process takes place, thus providing the basis for determining the adsorption capacity of bioadsorbent materials [24,25].

It is worth noting that current research is expanding toward unconventional sources for bioremediation, such as bacterial cellulose (a by-product of the beverage industry/kombucha fermentation) [24] and invasive *Sargassum* seaweeds [2]. While the use of thermally modified biomass for heavy metal adsorption is documented, previous studies typically employ pyrolysis temperatures between 400 and 800 °C. Furthermore, the raw and calcined states are frequently evaluated in isolation. This study determines the energetic threshold (calcination at 250 °C) required to modify the adsorption kinetics of Cr(VI). By providing a mechanistic comparison between the raw and thermally treated states, this work demonstrates that calcination at 250 °C unmasks the crystalline cellulose backbone and establishes a kinetic equilibrium time of 5 min. Consequently, this study evaluates the energy-to-efficiency ratio for biosorbent production.

## 2. Materials and Methods

### 2.1. Chemicals, Materials Collection, and Bioadsorbent Production

All reagents were of analytical grade and purchased from Merck KGaA (St. Louis, MO, USA), unless indicated otherwise. Raw peanut shells and orange peels were collected from local markets in the city of Queretaro, Mexico (20°35′27.6″ N, 100°23′27.6″ W). The samples were thoroughly washed with tap water to remove dust, soil, insects, and other extraneous matter. Following mechanical cleaning, the samples were sectioned into uniform pieces to facilitate subsequent dehydration. After washing, they were dried in an oven (Model FE-291, Felisa, Jalisco, Mexico) at 50 °C for two days, ground using a high-speed stainless steel blade mill (Cgoldenwall, Zhejiang, China), and sieved through a No. 100 mesh. Finally, they were subjected to thermal treatment in a furnace (BF51848C-1, Thermo Fisher Scientific, Waltham, MA, USA) at 250 °C for two hours with a ramp rate of 2.5 °C/min. Following this thermal modification, the calcined materials are strictly referred to throughout this manuscript as “biochars” (OP-C and PS-C), whereas the unmodified precursors are referred to as “raw bioadsorbents” (OP-R and PS-R). Both the raw bioadsorbents and the biochars were kept in sealed plastic bags and stored at 25 °C prior to characterization and adsorption experiments.

### 2.2. Characterization Methods of Bioadsorbents and Biochars

Thermogravimetric analysis (TGA) of the raw and biochars was conducted on a Q500 thermogravimetric analyzer (TA Instruments, New Castle, DE, USA). Approximately 10 mg of the sample was placed in a platinum sample pan and heated from 25 to 800 °C at a constant heating rate of 10 °C/min under a N_2_ atmosphere with a purge flow rate of 60 mL/min. Surface chemical functionality of the adsorbents were evaluated via Fourier-transform infrared (FTIR) spectroscopy in attenuated total reflection mode. The spectral profiles were obtained using a vector 33 FTIR spectrometer (Bruker, Ettlingen, Germany) equipped with a diamond crystal plate and recorded in a range of 4000–500 cm^–1^ at a spectral resolution of 4 cm^–1^ with 32 scans per spectrum. Specific surface area (SBET) and textural properties were determined via nitrogen physisorption isotherms at 77 K using an ASAP 2020 (Micromeritics, Nordcross, GA, USA) following 24 h of degassing at 120 °C. Crystalline phases and structural modifications of all samples were evaluated via X-ray diffraction (XRD). Powdered samples were packed into a standard aluminum holder and analyzed using a XRDynamic 500 diffractometer (Anton Paar, Graz, Austria) with a CuKα radiation (λ = 1.5406 Å, 40 kV, 49 mA). The diffractograms were recorded over a 2ϴ angular range from 4° to 60° with a continuous step size of 0.02°.

### 2.3. Adsorption Experiments

The 100 mg/L Cr(VI) stock solution was prepared by dissolving the required amount of potassium dichromate (K_2_Cr_2_O_7_) in deionized water. Adsorption experiments were carried out using an adsorbent dose of 0.5 g for each material: raw orange peel (OP-R), raw peanut shell (PS-R), calcined orange peel biochar (OP-C), and calcined peanut shell biochar (PS-C). The raw samples were used without any chemical or thermal treatment. Each bioadsorbent was placed in a beaker with 0.05 L of Cr(VI) solution at different concentrations (20, 30, 40, 50, and 60 mg/L) and magnetically stirred at 600 rpm using a digital stirring hotplate (Cimarec, Thermo Fisher Scientific, Waltham, MA, USA) for 120 min. The experiments were conducted at pH 4.0 and 25 °C. This specific pH was selected based on the speciation of Cr(VI) and the surface charge properties of the bioadsorbents and biochars. In aqueous solutions at pH values between 2.0 and 6.0, Cr(VI) exists predominantly as the monovalent hydrogen chromate anion (HCrO_4_^−^), which requires lower activation energy for adsorption compared to the divalent chromate ion (CrO_4_^2−^). Concurrently, at an acidic pH of 4.0—which is typically below the point of zero charge (pH_PZC_) for lignocellulosic biochars—the oxygen-rich functional groups on the biochar surface become protonated. This generates a net positive surface charge that promotes a strong electrostatic attraction toward the negatively charged HCrO_4_^−^ species, thereby optimizing the initial mass transfer and subsequent chemical interactions.

To determine the residual concentration of Cr(VI) in the solution, UV-vis spectra were measured (λ = 350 nm) using a Cary 5000 Varian spectrometer (Agilent Technologies, Santa Clara, CA, USA) [26].

The adsorption capacity (*q_e_*) and Cr(VI) removal (%) of the bioadsorbent materials were determined using Equations (1) and (2):

(1) $$q_{e}=\frac{C_{0}-C_{e}}{m}V$$

(2) $$\mathrm{Cr}(\mathrm{VI})\;\mathrm{removal}\;(\%)=\frac{C_{0}-C_{t}}{C_{0}}\times 100$$

where *C*_0_ (mg/L) is the initial concentration of Cr(VI), *C_e_* (mg/L) is the equilibrium concentration, *C_t_* (mg/L) is the final concentration of Cr(VI), *m* (g) is the dry mass of the sample, *V* (L) is the volume of the Cr(VI) solution, and *q_e_* (mg/g) is the adsorption capacity—specifically, the quantity of Cr(VI) ions adsorbed by the orange peel and peanut shell at equilibrium.

To evaluate the predominant kinetic mechanism and facilitate a direct comparison among the different raw and calcined materials, the kinetic modeling was strictly performed using the experimental data obtained at the highest initial Cr(VI) concentration (60 mg/L). This concentration was selected because it yielded the maximum adsorption capacity for all tested materials, allowing for a clearer evaluation of their optimal kinetic performance.

### 2.4. Adsorption Isotherm Fitting and Modeling Procedures

To better understand the mechanisms associated with Cr(VI) ion adsorption on the biochar surfaces, the experimental data obtained were modeled using the kinetic model equations shown in Table 1.

To evaluate the nature of the adsorption process and the interaction between Cr(VI) ions and the biochar surfaces, the experimental equilibrium data were analyzed using three established isotherm models: Langmuir, Freundlich, and Sips (Table 2). The Langmuir model assumes monolayer adsorption on a structurally homogeneous surface with energetically equivalent active sites, where no interaction occurs between adsorbed molecules. Conversely, the Freundlich model empirically describes multi-layer adsorption on heterogeneous surfaces with a non-uniform distribution of adsorption heat. Finally, the Sips model, a three-parameter composite isotherm, bridges the two approaches: it reduces to Freundlich behavior at low adsorbate concentrations and predicts a monolayer saturation capacity typical of the Langmuir model at high concentrations.

### 2.5. Statistical Analysis

All experiments were performed in triplicate to ensure the reproducibility and statistical reliability of the data. The experimental values reported represent the arithmetic mean ± standard deviation of the three replicates. Experimental data were fitted to mathematical models, and the goodness-of-fit and accuracy of the models were evaluated using the correlation coefficient (*R*^2^) and the reduced chi-square (*X*^2^) statistic. Higher values of *R*^2^ combined with lower values of *X*^2^ were used as the primary criteria to determine the best-fit model for the experimental data. All curve-fitting procedures and statistical analyses were performed using R Statistical Software version 4.5.2. (R Core Team 2021).

## 3. Results and Discussion

### 3.1. Characterization of Bioadsorbents and Biochars

**Figure 1:**
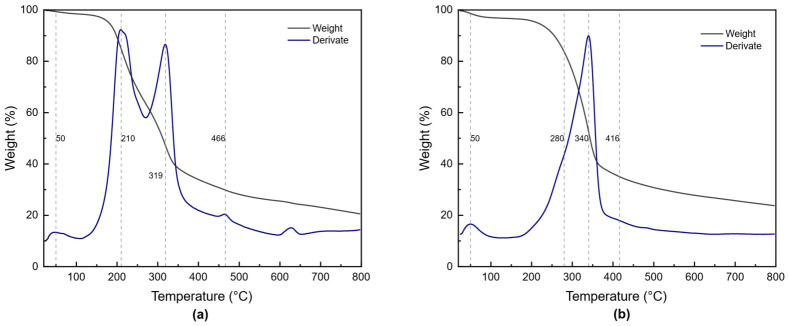
Thermogravimetric (TGA) and derivative thermogravimetric (DTG) curves of the raw biomass precursors: (**a**) orange peel and (**b**) peanut shell. The solid black line represents the weight loss percentage (%), while the solid blue line represents the rate of weight loss (derivative). Vertical dashed lines indicate the peak degradation temperatures corresponding to moisture evaporation, hemicellulose, cellulose, and lignin decomposition stages.

A thermogravimetric analysis of the orange peel and peanut shell materials was carried out to obtain the temperature at which the functional groups of the adsorbents decompose. The thermogravimetric (TGA) and derivative (DTG) curves for the raw precursors are presented in Figure 1. For the orange peel (Figure 1a), the DTG curve exhibits mass loss peaks at 210 °C, 319 °C, and 466 °C. As documented by Zapata et al. [32] in their thermo-kinetic analysis of orange peel, these thermal events are characteristic of the sequential decomposition of hemicellulose (∼215 °C), cellulose (∼310 °C), and lignin (>400 °C). For the peanut shell (Figure 1b), the degradation stages are observed at 280 °C, 340 °C, and 416 °C. Varma and Mondal [33] reported comparable pyrolytic pathways for peanut shell, noting that its structural rigidity shifts the cellulose degradation peak to higher temperatures compared to softer agricultural wastes. This behavior aligns with the thermal profiles of lignocellulosic materials established by Yang et al. [34], where amorphous hemicellulose decomposes at lower temperatures (220–315 °C) than crystalline cellulose (315–400 °C).

The thermal stability profile dictates the selection of the pyrolysis parameters. A treatment temperature of 250 °C was established for the modification of both materials. This temperature was selected because it lies between the decomposition onset of hemicellulose and the degradation peaks of cellulose and lignin, ensuring partial porosity development without carbon backbone collapse.

**Figure 2:**
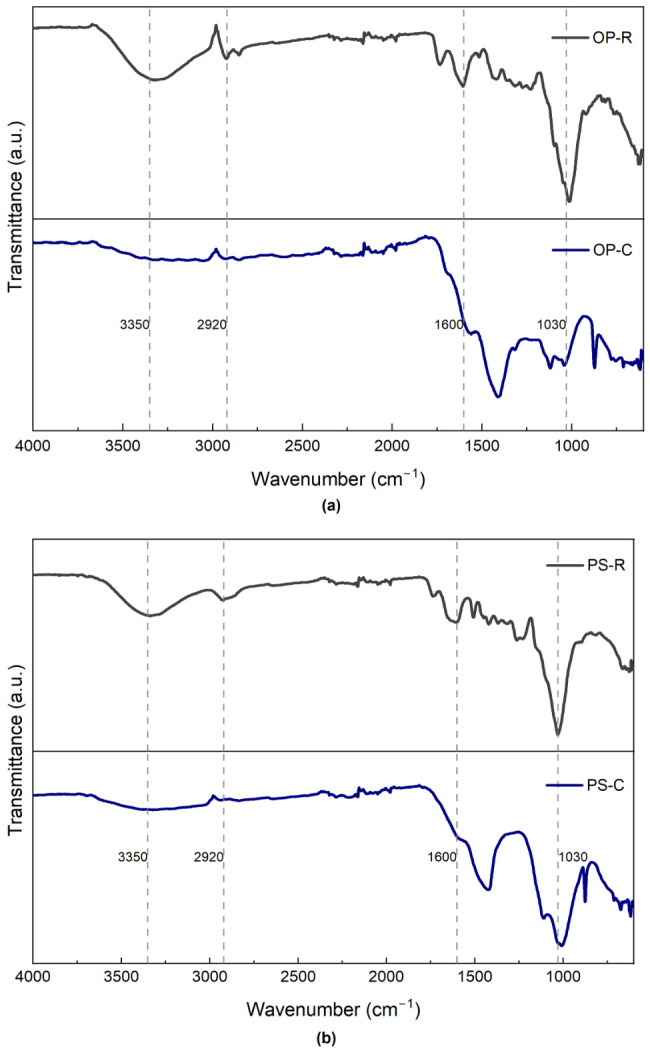
FTIR spectra of raw and calcined (250 °C) agroindustrial adsorbents: (**a**) orange peel (OP-R and OP-C) and (**b**) peanut shell (PS-R and PS-C). Vertical dashed lines indicate key vibrational modes associated with hydroxyl groups (∼3350 cm^–1^), aliphatic C-H stretching (∼2920 cm^–1^), aromatic C=C and carbonyl C=O groups (∼1600 cm^–1^), and C–O–C/C–O stretching in cellulose/hemicellulose (∼1030 cm^–1^).

Fourier transform infrared spectroscopy (FTIR) was performed to identify the functional groups of the adsorbent materials. The FTIR spectra of the raw and thermally treated materials, illustrated in Figure 2, reveal a thermochemical transformation. The decrease in the broad band at ∼3350 cm^–1^ (–OH stretching) and the peaks around 1030 cm^–1^ (C–O–C and C–O vibrations) in both OP-C and PS-C indicates the dehydration and partial degradation of the cellulosic and hemicellulosic fractions. Importantly, the retention of key oxygenated functional groups corroborates the structural integrity of the biochars after thermal treatment at 250 °C. As reported by Via et al. [35], the hemicellulose structural matrix is sensitive to thermal treatment, undergoing degradation near 250 °C.

Following degradation, the structural backbone remains intact. A shift to lower wavenumbers was observed in the band near 1600 cm^–1^ for the calcined materials in Figure 2a,b. According to Keiluweit et al. [36], this bathochromic shift indicates an aromatization process and an increase in the conjugation of C=C and C=O bonds within the lignin matrix. The preservation and structural rearrangement of these groups are directly related to the adsorption mechanism. While raw biomass primarily involves physical interactions, the modified materials possess functional groups that actively participate in Cr(VI) binding. As demonstrated by Guo et al. [37], these carboxyl and aromatic functionalities act as active sites that facilitate electrostatic attraction and reduction in Cr(VI). Specifically, the abundant hydroxyl (–OH) and carboxyl (–COOH) groups on the biochar surface function as primary binding sites and electron donors, directly driving the chemisorption of chromate anions. This establishes a direct link between the lignocellulosic composition of the raw precursors and the enhanced adsorption capacity of the materials.

While FTIR analysis qualitatively confirms the preservation and availability of these active functional groups following the 250 °C treatment, quantitative surface characterizations (such as Boehm titrations) are required in future studies to precisely determine the total acidic and basic surface site densities.

Nitrogen physisorption analysis was performed to determine the specific surface area (SBET), pore volume, and average pore diameter of the thermally modified materials, as summarized in Table 3.

As observed, the OP-C and PS-C exhibited specific surface areas of 1.06 m^2^/g and 10.43 m^2^/g, respectively. Although these values are relatively low compared to commercial activated carbons, this textural profile is highly characteristic and expected for biomass treated at mild pyrolysis temperatures (250 °C). At this specific thermal threshold, the lignocellulosic matrix undergoes initial dehydration and partial hemicellulose degradation. This degradation is associated with the development of a hierarchical mesoporous network without resulting in carbonization [36]. Low-temperature calcination represents a sustainable biorefinery strategy aimed at high recovery with low environmental impact [13], transforming organic matter into value-added products (biochar) by segregating lower-value compounds while stabilizing the material for industrial use. This structural transition induced an accessible mesoporous network, with average pore diameters of 30.63 nm for OP-C and 15.41 nm for PS-C. The combination of a relatively low surface area and wide mesoporous diameters suggests that Cr(VI) removal is not governed by physical trapping (physisorption) within a highly microporous labyrinth, which typically suffers from mass transfer resistance. Instead, the wide mesopores act as highly accessible channels, facilitating the diffusion of Cr(VI) ions directly toward the surface active sites.

**Figure 3:**
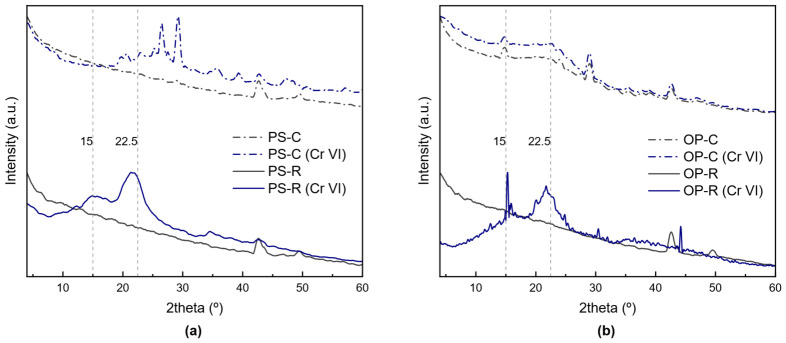
X-ray diffraction (XRD) patterns of the biomass-derived adsorbents before and after Cr(VI) interaction: (**a**) orange peel and (**b**) peanut shell systems. Solid lines represent the raw precursors (OP-R, PS-R) and their respective chromium-loaded counterparts (OP-R Cr(VI), PS-R Cr(VI)). Dashed lines indicate the thermally processed materials at 250 °C (OP-C, PS-C) and after the adsorption process (OP-C Cr(VI), PS-C Cr(VI)). Gray traces denote the baseline materials prior to adsorption, and blue traces correspond to the samples after Cr(VI) exposure. An explicit vertical offset separates the raw and calcined phases to isolate the thermal transitions.

Figure 3 presents the stacked XRD patterns for the orange peel (a) and peanut shell (b) systems, before and after Cr(VI) exposure. In the case of the orange peel precursor (Figure 3a), the uncalcined material prior to adsorption (OP-R, solid black line) exhibits a broad, featureless amorphous halo. This lack of defined reflections is attributed to the masking effect of the abundant amorphous pectin characteristic of citrus residues. The uncalcined orange peel subjected to the adsorption process (OP-R Cr(VI), solid blue line) displays the distinct emergence of massive reflections at 2θ ≈ 15° and 22.5°, corresponding to the (101) and (002) planes of native cellulose I [38]. This indicates that prolonged exposure to the aqueous medium during adsorption facilitates the partial dissolution and extraction of the soluble amorphous pectin matrix, thereby unmasking the insoluble crystalline cellulose backbone.

Following thermal treatment at 250 °C, the diffractograms of the modified materials (OP-C and OP-C Cr(VI), dashed lines) present an amorphous carbon profile, lacking the cellulose reflections. This confirms the thermal degradation of the lignocellulosic matrix at this temperature. The weak, sharp reflections observed in these calcined samples are attributed to native inorganic ash components and biominerals (e.g., calcium oxalates or carbonates) inherently present in the citrus peel, which become concentrated after the partial volatilization of the organic matter.

A parallel phenomenological behavior is observed in the peanut shell system (Figure 3b). The diffractograms of both the raw (PS-R) and thermally processed (PS-C) precursors prior to adsorption (black traces) exhibit predominantly amorphous halos. This indicates that the native crystalline cellulose domains remain heavily masked by the complex lignocellulosic matrix, primarily composed of amorphous lignin, hemicellulose, and extractives, which are not fully volatilized at the mild pyrolysis temperature of 250 °C [36].

However, upon exposure to the Cr(VI) aqueous solution (blue traces), the diffractograms of both PS-R Cr(VI) and PS-C Cr(VI) reveal the distinct emergence of the characteristic cellulose I reflections at 2θ ≈ 15° and 22.5°. Consistent with the orange peel system, this structural unmasking is attributed to the partial dissolution and leaching of water-soluble organic compounds (such as residual pectins and amorphous hemicellulose) during 120 min of continuous stirring in the aqueous adsorption medium, which consequently exposes the highly insoluble crystalline cellulose backbone.

As observed across all tested materials, the Cr(VI)-loaded spectra exhibit no sharp inorganic reflections corresponding to macroscopic chromium precipitates. This uniform crystallographic response suggests that the Cr(VI) uptake mechanism for both biomass-derived biochars is governed by surface interaction [37].

### 3.2. Adsorption Kinetics

**Figure 4:**
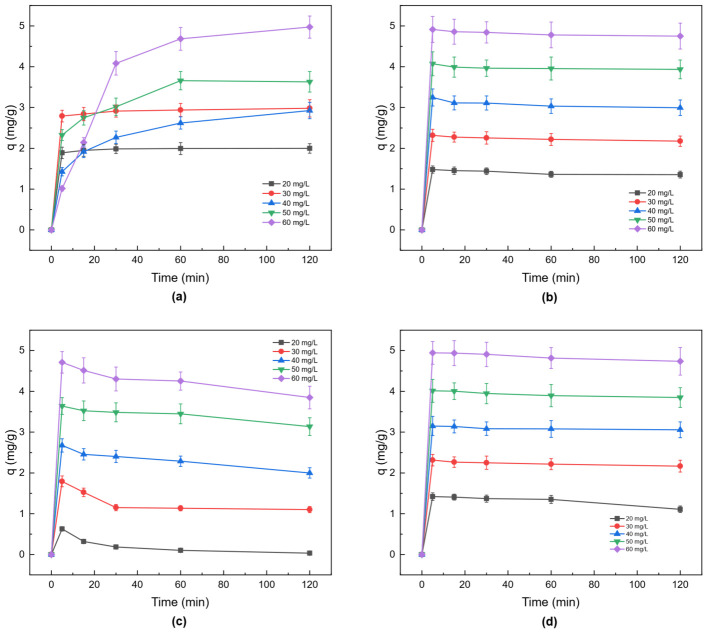
Effect of contact time and initial concentration on the Cr(VI) adsorption capacity at time *t* (*q_t_*) of (**a**) raw orange peel, (**b**) calcined orange peel biochar, (**c**) raw peanut shell, and (**d**) calcined peanut shell biochar.

Figure 4 shows the trend of Cr(VI) adsorption. In this figure, the evolution of adsorption capacity (*q_t_*) over time for the different materials under varying initial concentrations (20 to 60 mg/L) was evaluated to understand the mass transfer dynamics. Across all evaluated materials, an increase in the initial Cr(VI) concentration resulted in a proportional increase in adsorption capacity. This phenomenon is attributed to the fact that a higher concentration of ions in the solution increases the concentration gradient between the aqueous phase and the bioadsorbent surface, providing a greater driving force to overcome mass transfer resistance.

When comparing the materials derived from orange peel, a change in the saturation rate was evident. The raw peel (Figure 4a) required a longer contact time to stabilize at high concentrations, exhibiting a gradual adsorption curve. Conversely, the calcined orange peel (biochar) (OP-C, Figure 4b) reached adsorption equilibrium within the first 5 min of contact. The formation of a stable horizontal plateau after this time suggests a retention of Cr(VI), consistent with the high chemical affinity of the active sites exposed following the thermal treatment.

The most significant contrast was observed in the peanut shell biosorbents. The material in its raw state (PS-R, Figure 4c) presented anomalous behavior at high concentrations (50 and 60 mg/L), reaching an adsorption peak in the first few minutes followed by a continuous decrease in capacity (*q_t_*) over time. This drop suggests a desorption process, characteristic of weak and reversible physical interactions (physisorption) that fail to retain the adsorbate over prolonged periods.

However, the transformation of this agroindustrial waste into biochar via calcination completely suppressed this defect. The peanut shell biochar (PS-C, Figure 4d) reached capacities close to 4 mg/g without exhibiting any loss of adsorbed mass over time, suggesting that the structural modification induced an energetically stable binding primarily controlled by active site availability. The kinetic superiority and stability observed in the biochars compared to their raw counterparts corroborate the findings from the N_2_ physisorption analysis and the fit to the pseudo-second-order model. This hierarchical mesoporosity is fundamental to the adsorption performance observed in the kinetic studies. The development of mesopores and the increase in surface area following pyrolysis facilitate the access of Cr(VI) ions into the carbonaceous matrix, where they become anchored through surface interactions.

**Figure 5:**
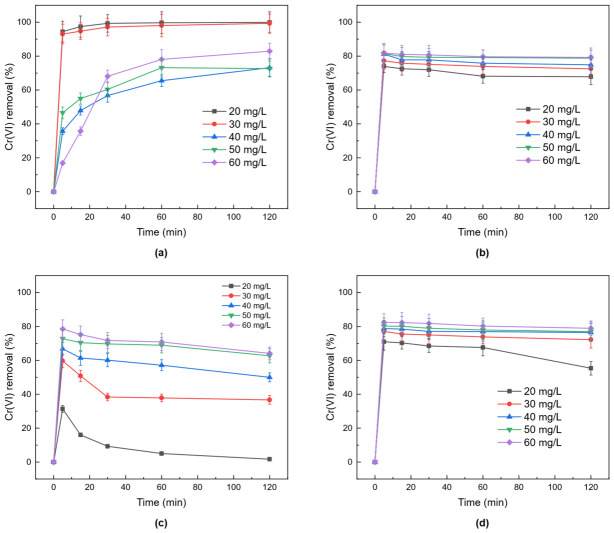
Comparison of Cr(VI) removal kinetics at varying concentrations (20–60 mg/L). Panels (**a**,**b**) illustrate the performance of orange peel before and after calcination, respectively, while (**c**,**d**) show the results for peanut shell.

The kinetic behavior of Cr(VI) removal for both adsorbents in their natural and calcined forms is presented in Figure 5. Figure 5a shows that the highest removal efficiency of Cr(VI) ions was achieved at 5 min for concentrations of 20 mg/L and 30 mg/L. This behavior is possibly due to a supersaturation effect of the ion concentration, which affected the removal time and efficiency, reflected in the kinetic constant values obtained for this material. At 120 min, the highest removal efficiency was achieved for concentrations of 40 mg/L, 50 mg/L, and 60 mg/L.

Figure 5b shows that the highest removal efficiency for all Cr(VI) concentrations was reached at 5 min and remained constant throughout the contact time. This can be attributed to the fact that the adsorption active sites were not yet saturated with the Cr(VI) concentration to which the material was exposed, generating constant removal behavior.

In Figure 5c, the highest removal percentage for all concentrations was obtained at 5 min, followed by a decay over time. This decay may be due to desorption of Cr(VI) ions from the material.

In Figure 5d, a similar behavior is observed for all concentrations, with greater efficiency at 5 min; subsequently, removal remained constant over time, with the exception of 60 mg/L, for which decay began at 60 min. Similar to calcined orange peel, this behavior can be attributed to the high quantity of available active sites, which allowed constant behavior.

**Figure 6:**
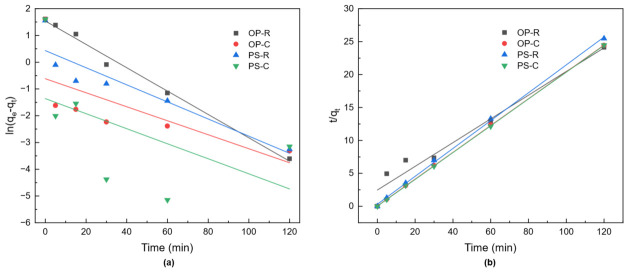
Linear plots of the (**a**) pseudo-first-order and (**b**) pseudo-second-order kinetic models for Cr(VI) adsorption onto the raw bioadsorbents and biochars. Symbols represent the experimental data evaluated at an initial concentration of 60 mg/L, and solid lines indicate the corresponding linear mathematical fits. Black symbols and lines correspond to raw orange peel (OP-R), red symbols and lines to calcined orange peel (OP-C), blue symbols and lines to raw peanut shell (PS-R), and green symbols and lines to calcined peanut shell (PS-C).

Table 4 summarizes the experimental kinetic constants (*k*_1_ and *k*_2_) and adsorption capacities (*q_e_*_1_ and *q_e_*_2_) as well as those obtained with the equation of the model used (*q_e_c*_1_ and *q_e_c*_2_) evaluated at an initial concentration of 60 mg/L. The graphical linear fits for both models are presented in Figure 6. In Table 4, it can be observed through *R*^2^ that the model which best fits the actual adsorption capacity values is the pseudo-second-order model; this is supported by the kinetic constant values which have a higher value than that obtained in the pseudo-first-order model.

The fit of the experimental data to the pseudo-second-order model (*R*^2^ > 0.99 for the calcined materials, as shown in Table 4) suggests that the rate-limiting step is likely controlled by the availability of active adsorption sites and surface occupancy. The notable increase in adsorption rate and equilibrium capacity (*q_e_*) for the calcined materials (OP-C and PS-C) can be directly attributed to the development of an accessible mesoporous network that overcomes the mass transfer resistance typical of raw biomass.

In the case of the raw peanut shell (PS-R, Figure 5c), anomalous behavior was observed with a decrease in removal after the initial 5 min. This suggests a desorption process, indicating weak and reversible physical binding (physisorption). This effect is not observed in the calcined materials, where chemisorption—an energetically more favorable and stable process—is dominant. This is reinforced by the value of the rate constant *k*_2_, which was significantly higher for the calcined materials (Table 4).

Table 5 compares the maximum adsorption capacity and equilibrium time of the calcined biochars with both recently reported agroindustrial waste-based adsorbents and advanced engineered materials for Cr(VI) removal. As observed, the adsorption capacity (*q_max_*) of the developed biochars (4.95 mg/g) is inherently lower than that of engineered synthetic adsorbents, such as anion exchange resins, activated carbons from industrial waste, complex magnetic nanocomposites, and other advanced composites (e.g., [39,40,41,42]). However, these advanced materials frequently demand complex, energy-intensive synthesis routes and the use of harsh chemical modifiers. The primary advantage of OP-C and PS-C lies in their kinetic efficiency. The mild 250 °C thermal activation employed in this study provides a kinetic alternative (reaching equilibrium in 5 min), demonstrating that agricultural residues can be transformed into kinetically efficient materials without the environmental and economic costs associated with advanced synthetic adsorbents.

As observed, while other modified biochars, such as brewery waste or rice straw, require between 420 and 480 min to reach equilibrium [43,44], and raw orange peel needs up to 90 min [45], the calcined biochars in this study (OP-C and PS-C) achieved complete equilibrium in 5 min. This performance represents a significant kinetic advancement even when compared to more advanced bio-based adsorbents [24]. For instance, functionalized bacterial cellulose (FBC)—recognized as a superior material due to its nanometric structure, high surface area, and exceptional purity—requires approximately 60 min to reach equilibrium for dye removal [24]. Furthermore, the kinetic efficiency of these calcined biochars far exceeds that of complex biomass-based systems like magnetic *Sargassum* composites, which, while highly effective for heavy metal recovery, necessitate up to 12 h (720 min) to reach stable chemisorption levels [2]. The kinetic superiority of OP-C and PS-C is directly attributed to the targeted low-temperature thermal modification. The calcination process at 250 °C successfully induced a hierarchical mesoporous network, achieving average pore diameters of 30.6 nm for OP-C and 15.4 nm for PS-C. These structural enhancements eliminated the physical barriers present in the raw bioadsorbents, exposed a higher density of active sites, and significantly reduced mass transfer resistance. Consequently, the kinetics and cooperative adsorption properties observed in these batch studies demonstrate the affinity of the biochars for Cr(VI) adsorption in aqueous environments. Overall, these results align with the chemisorption mechanism suggested by the kinetic data.

The current study evaluates fundamental adsorption kinetics and isotherms using synthetic monocomponent solutions. Future research must evaluate the interference of competing ions (e.g., sulfates, phosphates, nitrates) in multi-component aqueous systems to fully elucidate the adsorption selectivity of the developed biochars under variable matrix conditions.

### 3.3. Adsorption Isotherm Modeling

**Figure 7:**
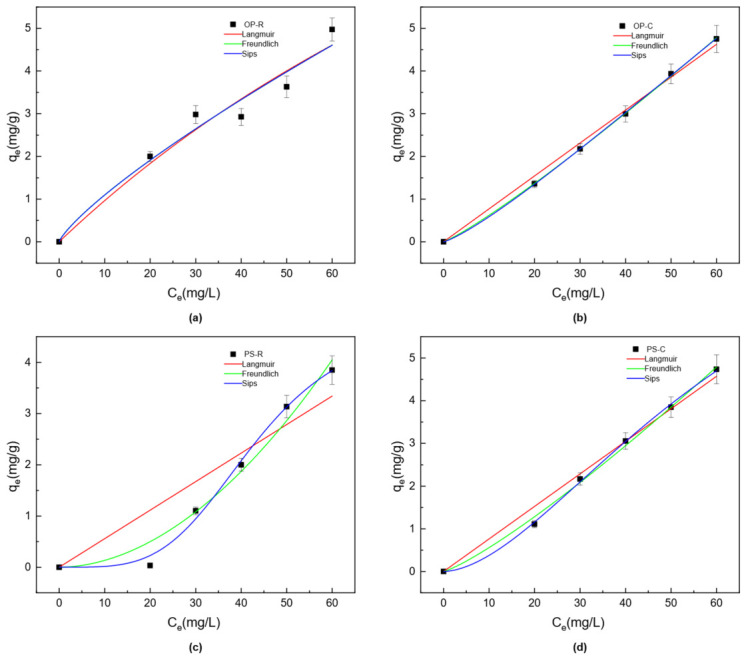
Experimental adsorption isotherms and mathematical model fits (Langmuir, Freundlich, and Sips) for Cr(VI) removal using: (**a**) raw orange peel (OP-R), (**b**) calcined orange peel biochar (OP-C), (**c**) raw peanut shell (PS-R), and (**d**) calcined peanut shell biochar (PS-C). Symbols represent the experimental equilibrium data, while the solid lines represent the corresponding model fits. In panel (**a**), the green line overlaps with the blue and red lines due to the close similarity of the model fits, making it visually indistinguishable.

To further elucidate the nature of the interaction between Cr(VI) ions and the biochar surfaces, equilibrium studies were performed. Figure 7 illustrates the experimental adsorption isotherms along with the fits for the Langmuir, Freundlich, and Sips models across the four tested materials.

A general increase in Cr(VI) removal is observed upon calcination (250 °C) for both precursors (orange peel and peanut shell). Crucially, none of the curves exhibit a distinct plateau, suggesting that the maximum saturation capacity has not been reached within the concentration range investigated (20–60 mg/L). To quantitatively distinguish the fitting performance of the models, the derived parameters and corresponding error functions are summarized in Table 6.

The equilibrium data for Cr(VI) adsorption were evaluated using non-linear regression for the Langmuir, Freundlich, and Sips models, with the resulting parameters and error functions (*R*^2^ and *χ*^2^) summarized in Table 6. The Langmuir model exhibited the poorest fit across all materials, yielding infinite standard errors for the theoretical maximum capacity (*q_m_*). These mathematically divergent and physically implausible values act as fitting artifacts, confirming that within the evaluated concentration range (20–60 mg/L), the biochars have not reached saturation, and adsorption does not occur via a strictly uniform monolayer. In contrast, the Freundlich and Sips models provided an excellent representation of the experimental data, presenting the lowest *χ*^2^ values.

**Figure 8:**
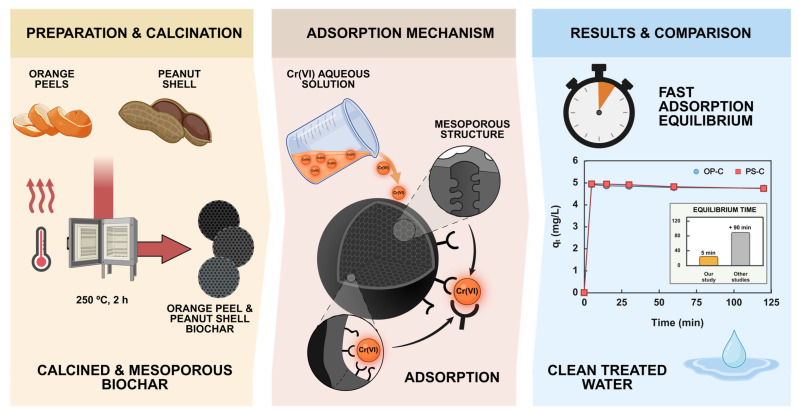
Schematic overview of the bioadsorbent preparation and the Cr(VI) remediation process. The diagram illustrates the conversion of raw orange peels and peanut shells into mesoporous biochars via low-temperature calcination (250 °C). The central panel details the proposed chemisorption mechanism, highlighting the diffusion of Cr(VI) ions into the newly developed mesoporous network and their subsequent adsorption by surface active sites. The right panel visually summarizes the primary empirical outcome of this structural modification: achieving an adsorption equilibrium in 5 min.

For the calcined materials (OP-C and PS-C), the Freundlich heterogeneity factor (*n*) was found to be less than one (0.878 and 0.833, respectively), while the Sips heterogeneity parameter (*β_S_*) exceeded one (1.225 and 1.743, respectively). These values are highly indicative of cooperative adsorption mechanisms on energetically heterogeneous surfaces. This cooperative multi-layer mechanism is a sophisticated response to surface heterogeneity, also observed in other functionalized polysaccharides where initial bonding creates active sites for continuous ion anchoring [24]. These thermodynamic findings strongly correlate with the structural properties demonstrated by the N_2_ physisorption analysis (Table 3). The thermal treatment successfully generated a significant mesoporous network, with average pore diameters of 30.6 nm for OP-C and 15.4 nm for PS-C. This degradation is associated with the development of the mesoporous network observed in the SBET analysis, without resulting in carbonization. This hierarchical mesoporosity not only increases the available active sites but also promotes cooperative interactions; once initial Cr(VI) ions are chemisorbed onto the high-energy sites of the biochar, lateral interactions facilitate the subsequent anchoring of additional ions. Consequently, the modified biochars act as efficient adsorbents, capable of continuously sequestering heavy metals through multi-layer accumulation within their mesopores without exhibiting early saturation (as conceptually summarized in Figure 8).

Furthermore, the investigated concentration range (20–60 mg/L) did not reach a saturation plateau, as evidenced by the mathematical divergence of the Langmuir model, meaning the absolute maximum adsorption capacity of the materials remains to be fully characterized. Finally, while the low-temperature treatment successfully induces a functional mesoporous network that drives rapid adsorption, the current study is limited to single-cycle batch equilibrium and kinetic thermodynamics. Future research must rigorously evaluate desorption cycles, adsorbate stability against leaching under varying pH and ionic strengths, and comprehensive life-cycle assessments to unequivocally confirm the long-term reusability and environmental sustainability of these agroindustrial biochars.

## 4. Conclusions

Low-temperature thermal modification (250 °C) of orange peel and peanut shell residues successfully yielded biochars with optimized physicochemical properties for the efficient remediation of Cr(VI) in aqueous solutions. This treatment proved pivotal in overcoming the inherent limitations of raw biomass, achieving adsorption equilibrium within just 5 min while effectively suppressing reversible physisorption and preventing contaminant desorption into the effluent. Isotherm modeling demonstrated that the adsorption process is best described by the Sips and Freundlich models. Specifically, the calculated Sips heterogeneity factor (*β_S_* > 1) suggests a cooperative multi-layer adsorption mechanism, which surpasses the restricted capacity of a uniform monolayer. This behavior is a direct result of the structural-thermodynamic synergy driven by the hierarchical mesoporous network developed during calcination, featuring average pore diameters of 30.6 nm for OP-C and 15.4 nm for PS-C. These mesopores function as highly active reservoirs where initial chemisorption of chromium facilitates the continuous anchoring of additional ions, thereby avoiding premature saturation of the material. While the kinetic and isotherm models suggest strong interactions and a rate-limiting step dependent on active site availability, future studies incorporating advanced spectroscopic techniques (e.g., XPS, zeta potential, and elemental mapping) are necessary to definitively elucidate the specific binding mechanisms and reduction states of chromium on the biochar surface. Ultimately, this study confirms that low-energy thermal treatment is a viable and cost-effective strategy to transform waste biomass into kinetically efficient adsorbents. The synergy between kinetics and cooperative mesoporous adsorption confirms that low-energy thermal modification effectively transforms these agricultural residues into acting materials for the efficient removal of Cr(VI) from aqueous solutions.

## Figures and Tables

**Table 1:** Adsorption kinetic models and parameters.

Kinetic Model	Linearized Equation	Parameters	Reference
Pseudo-first-order model	$ln\left(q_{e}-q_{t}\right)=ln\;q_{e}-k_{1}t$	*t* (min): contact time; *k*_1_ (min^–1^): pseudo-first-order rate constant; *q_t_* (mg/g): adsorption capacity at time *t*; *q_e_* (mg/g): equilibrium adsorption capacity	[27]
Pseudo-second-order model	$\frac{t}{q_{t}}=\frac{1}{k_{2}q_{e}^{2}}+\frac{t}{q_{e}}$	*k*_2_ (g/mg·min): pseudo-second-order rate constant	[28]

**Table 2:** Adsorption isotherm models and parameters used.

Isotherm Model	Equation	Parameters	Reference
Langmuir	$q_{e}=\frac{q_{m}K_{L}C_{e}}{1+K_{L}C_{e}}$	*C_e_* (mg/L): equilibrium concentration; *q_e_* (mg/g): equilibrium adsorption capacity; *q_m_* (mg/g): maximum monolayer adsorption capacity; *K_L_* (L/mg): Langmuir constant related to adsorption energy	[29]
Freundlich	$q_{e}=K_{F}C_{e}^{1/n}$	*K_F_* (mg/g)(L/mg)^1/*n*^: Freundlich affinity constant; *n* (dimensionless): adsorption intensity/heterogeneity factor	[30]
Sips	$q_{e}=\frac{q_{mS}K_{S}C_{e}^{\beta_{S}}}{1+K_{S}C_{e}^{\beta_{S}}}$	*q_mS_* (mg/g): maximum Sips adsorption capacity; *K_S_* (L/mg)^βS^: Sips equilibrium constant; *β_S_* (dimensionless): heterogeneity factor	[31]

**Table 3:** Textural properties of calcined orange peel and peanut shell.

Parameter	Calcined Orange Peel (OP-C)	Calcined Peanut Shell (PS-C)
Surface area (m^2^/g)	1.06	10.42
Pore volume (cm^3^/g)	0.00813	0.04017
Average pore diameter (nm)	30.63	15.41

**Table 4:** Kinetic models and constants of the materials used.

**Pseudo-First-Order Kinetic Constants**				
**Material**	***q_e_*_1_ (mg/g)**	***R*** **^2^**	***k*_1_ (min^−1^)**	***q_e_c*_1_ (mg/g)**
OP-R	5.00	0.9924	0.0437 ± 0.0019	4.6588 ± 0.1079
OP-C	4.95	0.4966	0.0262 ± 0.0132	0.5388 ± 0.7440
PS-R	4.75	0.8453	0.0320 ± 0.0068	1.5438 ± 0.3865
PS-C	4.95	0.2825	0.0281 ± 0.0224	0.2564 ± 1.2651
**Pseudo-second-order kinetic constants**				
**Material**	***q_e_*_2_ (mg/g)**	***R*** **^2^**	***k*_2_ (g/mg min^−1^)**	***q_e_c*_2_ (mg/g)**
OP-R	5.00	0.9644	0.0131 ± 0.9761	5.5568 ± 0.0173
OP-C	4.95	0.9999	0.6717 ± 0.0348	4.9159 ± 0.0006
PS-R	4.75	0.9992	0.1381 ± 0.1610	4.7308 ± 0.0028
PS-C	4.95	0.9999	2.2862 ± 0.0393	4.9186 ± 0.0007

**Table 5:** Comparison of Cr(VI) adsorption capacities and equilibrium times of various adsorbents.

Adsorbent	*q_max_* (mg/g)	Equilibrium Time (min)	Kinetic Model	Reference
Agroindustrial Waste-Based Adsorbents				
Brewers spent grain (BSG)	31.87	420	Pseudo-second	[43]
Rice Straw Biochar	10.03	480	Pseudo-second	[44]
Orange peel	4.96	90	Pseudo-second	[45]
Oil palm bagasse (OPB)	63.83	200	Pseudo-second	[46]
Sarkanda grass lignin	0.84	60	Pseudo-second	[47]
Mixture of coconut shell and coir (MCSC)	5.95	100	Pseudo-second	[48]
Mesoporous orange peel biochar (OP-C)	4.95	5	Pseudo-second	This study
Mesoporous peanut shell biochar (PS-C)	4.95	5	Pseudo-second	This study
Advanced/Engineered Adsorbents				
Activated mahogany fruit husk (MFHAC)	46.71	60	Pseudo-second	[39]
Activated carbon from used tyres	48.08	120	Pseudo-second	[40]
Anion exchange resin	208.92	85	Pseudo-second	[41]
Aminobiochar hydrogel (ABHG)	1250.00	180	Pseudo-first	[42]

**Table 6:** Non-linear isotherm parameters for Cr(VI) adsorption onto raw and calcined materials.

Isotherm Models	OP-R	OP-C	PS-R	PS-C
Langmuir				
*q_m_* (mg/g)	18.9578 ± 17.6533	40,249.70 ± 3.54 × 10^7^	131,509 ± 7.05 × 10^9^	44,291.2 ± 7.05 × 10^7^
*K_L_* (L/mg)	0.0053 ± 0.0062	1.91 × 10^−6^ ± 0.0017	4.23 × 10^−7^ ± 0.0112	1.7 × 10^−6^ ± 1.7 × 10^−6^
*R* ^2^	0.9569	0.9944	0.8496	0.9859
*χ* ^2^	0.1500	0.0210	0.4807	0.0540
Freundlich				
*K_F_* (mg/g) (L/mg)^1/n^	0.1732 ± 0.1035	0.0452 ± 0.0028	0.0016 ± 0.0018	0.0353 ± 0.0083
*n*	1.247 ± 0.2456	0.878 ± 0.0125	0.524 ± 0.0784	0.833 ± 0.0427
*R* ^2^	0.9612	0.9997	0.9726	0.9966
*χ* ^2^	0.1350	9.9 × 10^−4^	0.0873	0.0131
Sips				
*q_mS_* (mg/g)	507.93 ± 16.0224	43.81 ± 39.6432	4.91 ± 0.6915	9.96 ± 2.302
*K_S_* (L/mg)^βS^	3.38 × 10^−4^ ± 1.05 × 10^−5^	8.07 × 10^−4^ ± 5.39 × 10^−4^	4.03 × 10^−7^ ± 1.17 × 10^−6^	7.09 × 10^−4^ ± 2.84 × 10^−4^
*β_S_*	0.8050 ± 1.38 × 10^−4^	1.2250 ± 0.0855	3.9060 ± 0.85	1.7430 ± 0.1943
*R* ^2^	0.9999	0.9998	0.9946	0.9990
*χ* ^2^	1.28 × 10^−6^	9.7 × 10^−4^	0.0228	0.0048

## Data Availability

The original contributions presented in this study are included in the article. Further inquiries can be directed to the corresponding authors.
